# A Physio-Morphological Trait-Based Approach for Breeding Drought Tolerant Wheat

**DOI:** 10.3389/fpls.2020.00715

**Published:** 2020-06-03

**Authors:** Kamal Khadka, Hugh J. Earl, Manish N. Raizada, Alireza Navabi

**Affiliations:** Department of Plant Agriculture, University of Guelph, Guelph, ON, Canada

**Keywords:** drought tolerance, physiology, morphology, high throughput phenotyping, wheat, climate change, traits, breeding

## Abstract

In the past, there have been drought events in different parts of the world, which have negatively influenced the productivity and production of various crops including wheat (*Triticum aestivum* L.), one of the world’s three important cereal crops. Breeding new high yielding drought-tolerant wheat varieties is a research priority specifically in regions where climate change is predicted to result in more drought conditions. Commonly in breeding for drought tolerance, grain yield is the basis for selection, but it is a complex, late-stage trait, affected by many factors aside from drought. A strategy that evaluates genotypes for physiological responses to drought at earlier growth stages may be more targeted to drought and time efficient. Such an approach may be enabled by recent advances in high-throughput phenotyping platforms (HTPPs). In addition, the success of new genomic and molecular approaches rely on the quality of phenotypic data which is utilized to dissect the genetics of complex traits such as drought tolerance. Therefore, the first objective of this review is to describe the growth-stage based physio-morphological traits that could be targeted by breeders to develop drought-tolerant wheat genotypes. The second objective is to describe recent advances in high throughput phenotyping of drought tolerance related physio-morphological traits primarily under field conditions. We discuss how these strategies can be integrated into a comprehensive breeding program to mitigate the impacts of climate change. The review concludes that there is a need for comprehensive high throughput phenotyping of physio-morphological traits that is growth stage-based to improve the efficiency of breeding drought-tolerant wheat.

## Introduction

Wheat (*Triticum aestivum* L.) is ranked second among cereal crops in terms of total global production but ranked number one in terms of the total area under cultivation (FAO, 2018a; OECD-FAO, 2018). The global annual production of wheat in 2017 was 757 million metric tons (FAO, 2018a). Globally, wheat is responsible for 41% of total cereal calorie intake, broken down as 35 and 74% in developing and in developed countries, respectively (Shiferaw et al., 2013). Currently, wheat ranks second after rice in terms of dietary intake volume, with 68% of the wheat produced used for food, and approximately 19% for feed, and the rest for other purposes, including industrial biofuels (FAO, 2018b). Imports of wheat in developing countries, including in the tropics where wheat is not grown, are increasing (FAO, 2018b). For example, 2–3% increases in wheat demand per year have been observed in Sub-Saharan Africa (CIMMYT, 2017). In particular, Sub-Saharan Africa imports wheat not only because of rapid population growth but also due to biotic and abiotic factors that constrain crop production, in addition to climate change, changing food habits of local people (Tadesse et al., 2018) and an inability of farmers to cope with market fluctuations and price shocks for different crops (Antonaci et al., 2014). Global hunger has increased in the past 3 years (FAO, 2018c), and the current data reveals that we are not in a situation to eradicate hunger by 2030 as targeted by sustainable development goals. The demand for wheat is expected to increase by 60% to feed the human population which is expected to surpass nine billion by 2050; to achieve this, there is a need to accelerate global average wheat yield increases from the current 1% per year to a minimum of 1.6% (GCARD, 2012).

Losses in wheat production are mainly due to abiotic factors including drought, salinity, and heat stress rather than biotic factors (Abhinandan et al., 2018). Unfortunately, recurrent drought events have threatened global wheat production which necessitates major attention. The effect of water stress differs at different growth stages of wheat (Daryanto et al., 2016) while the duration and intensity of water stress can affect the development of wheat at different trait levels (Sarto et al., 2017) which ultimately reduces grain yield. Various reports from around the world indicate that limited water availability plays a major role in reducing wheat yield. According to FAO (2018c), global wheat production in 2018 was predicted to decline by 2.7% which is based on predictions of changing weather. A meta-analysis of 60 published studies showed that drought reduced wheat yields by an average of 27.5% (Zhang J. et al., 2018), and a similar study, which included peer-reviewed articles from 1980 to 2015, showed decreases of 20.6% (Daryanto et al., 2016). For example, in Australia, wheat productivity has been severely affected by water stress due to drought events among many other factors (Curtis and Halford, 2014). Specifically, there were severe drought events in Australia during 1982, 1994, 2002, 2004, and 2006, which resulted in yield reductions of five major field crops including wheat by 25–45% compared to the years with optimum rainfall (Madadgar et al., 2017). In this region, the average wheat yield at field sites was only around 0.8 t/ha due to severe drought over the 2006 crop season (Fleury et al., 2010). Between 2005 to 2007, Australian food prices increased at twice the rate of the consumer price index, and this increase was attributed to the drought events of 2004 and 2006 (Quiggin, 2007). Similarly, inconsistencies in winter wheat production were observed during the 2011 to 2013 drought in Texas, United States: a yield reduction was observed in 2011 and 2014 while the yield increased in 2012 (Ray et al., 2018). Many African countries have also been prone to drought events at different times of the year, including a 2009 drought in Kenya which reduced wheat production by 45% (Rauf et al., 2016).

**FIGURE 1:**
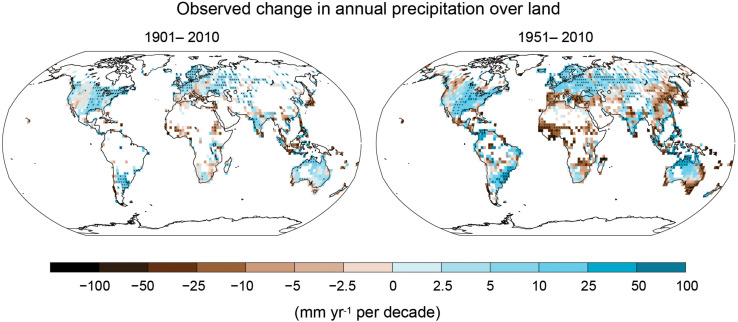
Global map showing the change in precipitation observed during two different time periods from 1901 to 2010 **(left)** and 1951 to 2010 **(right)**. The trends were calculated only for the grid boxes indicated on the maps which had >70% complete data records and more than 20% data availability for the first and last 10% of the time period. Incomplete data sets are indicated by white areas while significant trends are indicated by a black plus sign (+). Source: IPCC, 2013.

Different climate change studies have developed models that predict changes in the frequency and intensity of precipitation, increases in global temperatures, and a rise in atmospheric CO_2_ concentrations (Rosenzweig and Parry, 1994; Mahato, 2014). Figure 1 shows the change in annual precipitation over the last century. The trend from such historical observations and future climate change models indicates that some regions will receive more precipitation while others will get drier (IPCC, 2013). In general, it is expected that changes in the global climate will have both spatial and temporal impacts on agricultural production (Kulukulasuriya and Rosenthal, 2003).

**FIGURE 2:**
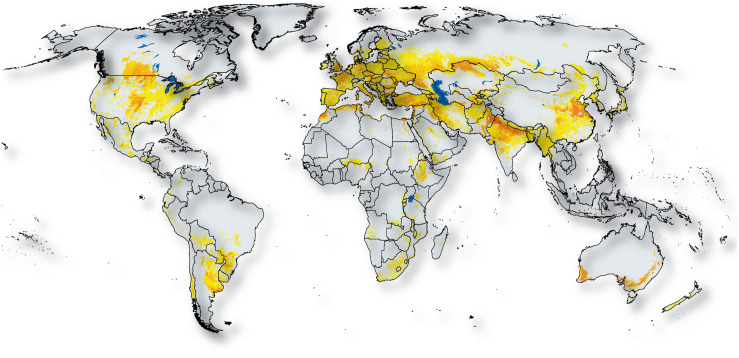
Major wheat growing areas around the world. Darker colors show regions where more wheat is grown. Map based on You et al. (2014). (Source: wheat.org).

Some of the major wheat growing areas (Figure 2) are going to be profoundly affected by drought in the years to come, specifically in South Eastern and South Western Australia, parts of Western China, the Indo-Gangetic plains, the Middle-East, Southern Europe, and Western Canada. A recent study (Daryanto et al., 2016) analyzed wheat drought data from 144 studies around the world, published between 1980 and 2015, and showed that drought conditions caused a 20.6% average decrease in wheat yield corresponding to a 40% reduction in water availability at the global level. Similarly, the Punjab and Haryana States in the Indo-Gangetic plain experienced a prolonged duration of low wheat yield from 2002 to 2010, mainly attributed to depleted groundwater resulting from an insufficient monsoon, poor surface water irrigation and higher temperature (Figure 1; Mukherjee et al., 2019). In China, a study using integrated climate assessment models estimated a ∼55% yield loss in wheat yield due to drought during 1955–2014 compared to a ∼7% yield loss under the baseline irrigation scenario (Yu C. et al., 2018). The same study also predicted that the yield loss rate will double based on a current 100-year drought projection for rain-fed environments. Combined, these examples suggest that drought is going to be a critical yield limiting factor for wheat in some wheat growing areas of the world in the years to come.

There are a limited number of comprehensive studies that quantify the impacts of drought on agriculture because of the complex nature of drought and its complicated association with other abiotic factors such as heat or the soil profile type (Nicolas et al., 1984; Ali et al., 1999; Eriyagama et al., 2009). Drought can be divided into three broad inter-related groups: meteorological drought, caused by anomalies in the atmosphere and higher temperatures; agricultural drought, caused by low precipitation and high evapotranspiration rates; and hydrological drought when sources of water fall below their normal average (Dai, 2011). Drought conditions may be defined as mild, moderate or severe drought based on the duration and extent of water availability. However, there is inconsistency in the use of such classifications due to confounding factors such as the soil type and the experimental environment (i.e., greenhouse or field). For example, in a greenhouse experiment intended to evaluate wheat seedling traits under water limitation, Ahmed et al. (2019) defined optimal water as 100% field capacity and stress as 50% field capacity. By contrast, in a field experiment involving rainout shelters, severe, moderate and optimal water conditions for wheat were defined as 35–40%, 55–60%, and 75–80% field capacity, respectively (Abid et al., 2018).

It is clear that drought negatively affects crop growth, development, dry matter production, and potential yield (Zhang et al., 2006; Anjum et al., 2011; Zhang J. et al., 2018). Grain yield is the basis for selection in most breeding programs for drought tolerance. However, grain yield is affected by many factors aside from drought. From a molecular perspective, drought tolerance is a very complex trait involving many drought-responsive genes that differ in expression at different growth stages (Blum, 2011), each of which generally makes a minor contribution to the trait (Sallam et al., 2019). Pyramiding their additive gene action by crossing complementary drought tolerance traits from different growth stages may achieve greater results than direct selection on yield alone. Additionally, selection using earlier proxy traits may be more time efficient than yield. In general, as reviewed below, the extent to which drought impacts plant growth and physiology largely depends upon the growth stage at which it is exposed to drought and the plant species/genotype (Kondo et al., 2004).

The first objective of this review is to describe growth-stage based physio-morphological traits that can be targeted by breeders to develop drought-tolerant wheat varieties. The second objective is to describe advances in precision phenotyping of drought tolerance related physio-morphological traits under field conditions. Such phenotyping is required to reveal the genetic basis of these traits which are complex and quantitative (i.e., many minor quantitative trait loci, QTLs) (Fleury et al., 2010; Tuberosa and Maccaferri, 2015) as already noted.

There have been related reviews on these subjects. In particular, Monneveux et al. (2012) highlighted the methodological approach for the use of physiological traits in breeding for drought tolerance in wheat, while Sallam et al. (2019) reviewed a wide array of studies pertaining to drought physiology in plants along with advances in wheat breeding for drought tolerance. In this review, we focus on more practical aspects of wheat breeding by focusing on specific physio-morphological traits at different growth stages that are affected by moisture stress. These stage-specific traits may be potential targets for future selection. This paper also highlights advances in phenotyping of these traits and concludes with a comprehensive strategy for breeding drought tolerant wheat by taking into account these approaches.

## Targets for Breeding Drought-Tolerant Wheat

### Selection Environment

Considering the dynamic nature of abiotic stresses, the empirical approach to breeding for drought tolerance emphasizes selection under both optimal and stressed conditions to observe yield stability and yield potential (Bruckner and Frohberg, 1987). Plant breeders have been adopting replicated, multi-location, and multi-year variety testing to identify varieties that perform best across a wide range of environments as part of empirical breeding based programs. If the objective is to breed a crop to tolerate a specific stressful environment, then direct selection under such an environment results in higher stability and durability of the crop yield (Ceccarelli, 1987; Ceccarelli et al., 1998). This observation was supported earlier by Johnson and Frey (1967) who reported that varieties selected directly from stressed conditions exhibit a low genotype × environment (G × E) interaction compared to those selected under optimal conditions. However, some studies show that selection under optimal environments also improves grain yield under drought conditions to a limited extent (Araus et al., 2002; Cattivelli et al., 2008; Sserumaga et al., 2018). Mohammadi and Amri (2011) indicate that grain yield improvement may be attained by either selection in low input and stressed environments or by selection in non-stressed conditions followed by selection under stressed conditions. Practically, drought stress may not occur every season. Therefore, the evaluation of traits only under stressed conditions may limit the variety development process by losing potential genetic materials that perform better in a normal wheat-growing environment. Furthermore, a variety that performs better under different environmental conditions may be more stable in terms of grain yield across different years and environments. Therefore, employing a wide range of testing environments including both normal and stressed could be more appropriate and efficient for the development of high yielding, stable varieties adapted to drought-prone environments.

### Physio-Morphological Traits

Advances in precision phenotyping, along with combining genetic and molecular approaches in the breeding process, are expected to improve the efficiency of breeding programs (Mir et al., 2012; Kosová et al., 2014; Choudhary et al., 2018). In this scenario, indirect selection that targets the underlying physiological traits that contribute to yield can be more efficient than direct selection for higher yield (Reynolds et al., 2005; Reynolds and Trethowan, 2007). The reason for this observation is that traditional yield-based breeding relies on yield analysis of tens of thousands of plants at the end of each breeding cycle which in general masks the effect of the trait of interest on grain yield. By contrast, the hope of physio-morphological trait-based breeding is that early season and/or simple surrogate traits can be identified for yield or yield attributing traits (Nigam et al., 2005). This means that the measurement of yield attributing physio-morphological traits independent of grain yield improves the efficiency of selection by reducing the reliance on final grain yield. This approach may increase the possibility of making more successful crosses in a breeding program by exploiting the potential for additive gene action (Reynolds et al., 2009a; Ataei et al., 2017; Dolferus et al., 2019), as already noted above. In addition, it is always an advantage if the physiological trait considered for selection under a harsh environment has heritability higher than yield itself, which confers a greater chance for success for the development of a stress tolerant variety.

## Growth Stage Based Targets

**FIGURE 3:**
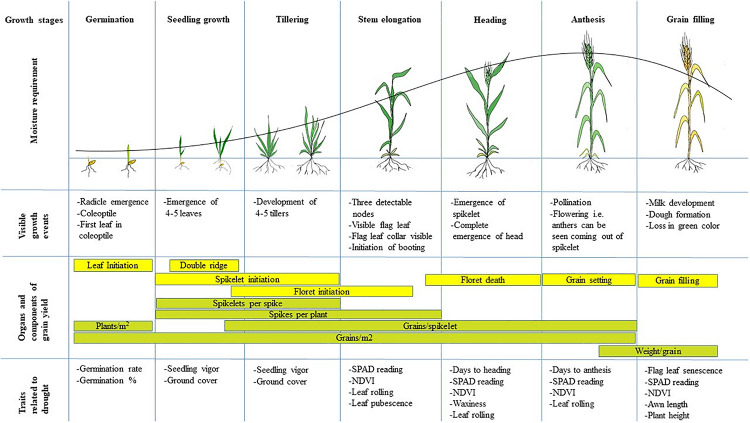
The figure shows different growth stages of wheat along with the associated visible growth events and traits related to drought tolerance. Also shown are the components that contribute to final grain yield that are important during each growth stage. Adapted from Slafer and Rawson (1994). The line graph shows the trend of moisture requirements at different growth stages.

Increasing the efficiency of breeding drought-tolerant wheat varieties by targeting physio-morphological traits requires a thorough understanding of the impact of drought at different growth stages. In general, while the intensity and frequency of drought are extremely critical to the overall performance of the crop, the phenological stage at which drought events occur is equally important (Sarto et al., 2017). Wheat plants may be more susceptible to drought at specific critical growth stages, i.e., germination and seedling stages (Akram, 2011); tillering and stem elongation stages (Saeidi et al., 2015; Wang et al., 2015; Ding et al., 2018); and heading, anthesis and grain filling stages (Akram, 2011; Sarto et al., 2017). The morphological traits that contribute to final grain yield differ at each growth stage, and the extent to which these are impacted by drought, determine the seriousness of the stress event (Figure 3). Long term droughts such as those starting from stem elongation through to maturity, reduce yield more significantly compared to those starting at later phases through to maturity (Shamsi and Kobraee, 2011).

Combined, these observations suggest the need to understand the crop growth stages of wheat, the intensity of water stress at any given growth stage, and also the timing and extent of drought stress in a given target environment, to develop a drought-tolerant variety for any specific environment. The following sub-sections detail the effect of drought at different growth stages of wheat as well as drought tolerance-related physio-morphological traits critical to developing drought-tolerant wheat varieties.

It is important to note, however, that severe drought at any phenological stage of wheat may potentially reduce the final grain yield. For example, a study that explored drought tolerance of ten wheat genotypes under different levels of stress treatments showed that all the growth stages were influenced by limited moisture availability (HongBo et al., 2005). More recently, Ihsan et al. (2016) also observed that multiple growth stages, including germination, tillering, booting, heading, anthesis, and maturity, are negatively affected by drought stress, although the effect on heading and grain filling stages was more severe.

It is important to note that in the literature (e.g., Table 1), the severity of drought (mild or severe) that is reported can be subjective, dependent on the authors of a particular study. For example, Ding et al. (2018) defined treatments with 75, 60, and 40% soil moisture content as control, mild drought and severe drought, respectively, while other studies have used different thresholds (Table 1). It may be more useful to define drought severity objectively, based on the quantitative frequency and duration of drought stress. In any case, prolonged mild, moderate or severe stress will increasingly reduce final grain yield.

**TABLE 1:** Examples of previous studies that measured wheat yield declines due to drought imposed at different growth stages.

**Growth stages**	**Stress level^§^**	**Grain Yield reduction**	**Notes**	**Source^**
Germination and seedling stages	Moderate	7%	• One variety • Water withheld for 7 days at spikelet initiation stage (3 leaf stage) • Rapid recovery after re-watering	Zhang et al., 2013
Tillering	Severe Moderate	6–16% 2–13%	• One drought tolerant and one drought susceptible variety: 6% vs. 16% reduction (severe stress), 2% vs. 13% reduction (moderate stress) • Drought treatments were 10 days at 35–40% (severe) and 55–60% (moderate) vs. 75–80% (control) field capacity • Drought tolerant variety had small yield reduction by maintaining high photosynthetic rate during drought and had rapid recovery	Abid et al., 2018
	Severe	52%	• Yield averaged over 10 varieties • Water withheld during tillering (duration not reported) • Number of grains significantly reduced	Mehraban et al., 2019
	Moderate	4–13%	• Three local varieties • Water withheld at up to 50% of the optimal gravimetric soilwater content (44.32 g H_2_0/25 Kg dry soil) • Recovery after re-watering	Maqbool et al., 2015
Stem elongation	Severe Mild	53% 0%	• One variety • Relative soil moisture content at 40% (severe drought), 60% (mild drought) and 75% (control) • Decrease in spikes per plant and kernel weight • Drought reduced kernel number, but compensated by increased kernel weight for mild stress	Ding et al., 2018
	Severe Moderate	15–24% 5–11%	• One drought tolerant and one drought susceptible variety: 15% vs. 24% reduction (severe stress), 5% vs. 11% reduction (moderate stress) • Drought treatments were 10 days at 35–40% (severe) and 55–60% (moderate) vs. 75–80% (control) field capacity • Yield observations noted above	Abid et al., 2018
	Severe	2–45%	• Five varieties including 2 drought tolerant varieties • Water withheld for 7–10 days: volumetric water content (v/v %) 3.5% (drought) vs. 20–25% (control). • Reduced kernel number	Varga et al., 2015
Stem elongation to anthesis	Severe	54%	• Yield averaged across four varieties • Treatments were 50% (drought) vs. 100% (control) field capacity • Number of grains per spike	Saeidi et al., 2015
	Severe Moderate Mild	11–3% 7–10% 0–4%	• One drought tolerant and one drought susceptible variety: 11% vs. 13%, 7% vs. 10% 0% vs. 4% reductions for severe, moderate and mild stress, respectively • Drought treatments were 40–45% (severe), 55–60% (moderate), 65–70% (mild) vs. 75–80% (full irrigation) of field capacity • Reduced canopy photosynthesis and translocation	Liu et al., 2016
Booting, heading and anthesis	Severe	46–82%	• Six varieties • Grown at 100% field capacity then water withheld for 20 days (drought) after booting and anthesis • Reduction in kernel number	Khakwani et al., 2012
	Severe	47%	• Yield averaged across three varieties • Water withheld after the onset of the growth stage (duration not reported) • Reduced kernel number and kernel weight	Shamsi et al., 2010
Heading	Severe Mild	38% 11%	• One variety • Drought treatments were: relative soil moisture content maintained at 40% (severe), 60% (mild) vs. 75% (control) • Reduction in number of spikes per plants, kernel weight and number	Ding et al., 2018
	Severe	25-78%	• Five varieties including 2 drought tolerant varieties • Water withheld for 7–10 days: volumetric water content (v/v %) 3.5% (drought) vs. 20–25% (control). • Reduced kernel weight and number	Varga et al., 2015
Anthesis	Severe	69%	• Yield averaged across 4 synthetic hexaploids and 2 standard checks • Water withheld for 16 days • Reduction in kernel number and grain weight	Pradhan et al., 2012
	Moderate	11%	• One variety • Water withheld for 6 days just after heading • Slight reduction in kernel number per spike	Zhang et al., 2013
	Moderate	19–42%	• Three local varieties • Water withheld at up to 50% of the optimal gravimetric soil water content (44.32 g H_2_0/25 Kg dry soil) • Reduced number of kernels per spike	Maqbool et al., 2015
Grain filling	Severe	57%	• Yield averaged across 5 varieties • Drought reported as 40–45% of the natural water content vs. 60–70% for the control, starting 12 days after heading (duration not reported) • Significant reduction in kernel weight and number	Balla et al., 2011
	Severe	24–87%	• Five varieties including 2 drought tolerant varieties • Water withheld for 7–10 days: volumetric water content (v/v %) 3.5% (drought) vs. 20–25% (control). • Reduced kernel weight	Varga et al., 2015
	Severe	15%	• Yield averaged over 10 varieties • Water withheld during grain filling (duration not reported) • Reduced kernel weight	Mehraban et al., 2019
	Severe	31%	• Yield averaged across three varieties • Water withheld after the onset of grain filling stage (duration not reported) • Reduced kernel weight	Shamsi et al., 2010
	Severe	26%	• Yield averaged across 4 synthetic hexaploids and 2 standard checks • Water withheld for 21 days after anthesis • Kernel weight reduced	Pradhan et al., 2012
	Severe	28.2%	• Yield of one variety averaged over 2 years • Drought imposed by rain-out shelter after anthesis until maturity; control was rainfed (∼47 and 95 mm rainfall in 2 years) • Reduction in kernel weight and number	Gevrek and Atasoy, 2012
	Severe Moderate Mild	13–3% 7–12% 0–1%	• One drought tolerant and one drought susceptible variety: 13% vs. 13%, 7–12%, 0–1% reductions for severe, moderate, and mild stress, respectively • Drought treatments were 40–45% (severe), 55–60% (moderate), 65–70% (mild) vs. 75–80% (full irrigation) of field capacity • Reduced canopy photosynthesis and translocation	Liu et al., 2016
	Moderate	24–48%	• Three local varieties • Water withheld at up to 50% of the optimal gravimetric soil water content (44.32 g H_2_0/25 Kg dry soil) • Reduced kernel weight	Maqbool et al., 2015

*^§^The criteria used to define the severity of drought stress was defined by each study and there is inconsistency in this definition between studies. ^All were greenhouse/control environment trials except for Shamsi et al. (2010), Gevrek and Atasoy (2012), Liu et al. (2016), and Mehraban et al. (2019) which were under field conditions.*

### Germination and Seedling Stages

With respect to the impacts of drought at specific growth stages in wheat, it is known that sufficient moisture in the soil, along with optimum temperature, is required for uniform germination and hence may be critical for drought-sensitive varieties (He et al., 2017; Mukherjee et al., 2019). This is because germination-related traits such as emergence index, emergence rate index, the energy of emergence, and relative cell injury (RCI) vary significantly among different wheat varieties under normal and water-limited conditions (Ahmad et al., 2015). Increasing the level of stress during the germination and early seedling phases negatively affects traits such as germination rate, seedling vigor, and lengths of coleoptile, shoot and/or root (Kızılgeçi et al., 2017). There are limited studies that quantify yield losses in wheat due to drought at the germination and seedling stages. However, a few studies have reported a positive association of seedling traits with reproductive traits including grain yield (Kandic et al., 2009; Dodig et al., 2015). These studies highlight the importance of seedling drought tolerance to final plant performance.

### Tillering and Stem Elongation Stages

After double ridge formation, spikelet initiation begins right at the seedling stage and proceeds until the tillering stage, while floret initiation starts at tillering and continues during the stem elongation period. Thus, these growth stages are important for maintaining the spikelet number per plant and spikes per plant which directly contribute to grain yield. As a result, a severe drought imposed during tillering and stem elongation in wheat reduces the number of grains per spike and ultimately grain yield (Blum et al., 1990; Saeidi et al., 2015; Ding et al., 2018; Table 1). For example, Saeidi et al. (2015) observed a 54% reduction in grain yield as a result of water stress at the vegetative stage (stem elongation to flowering). In one study Ding et al. (2018) observed that extreme water stress during the stem elongation period reduced grain yield by up to 72%, which was greater than the effect of extreme water stress during the reproductive period. Similarly, in another study, the greatest decrease in grain yield observed as a result of moisture stress was at the stem elongation stage compared to booting and grain filling stages (Keyvan, 2010). In addition to declines in stem growth and the number of effective tillers, plant height is also reduced by drought at this stage (Blum et al., 1990; Sarto et al., 2017), and overall plant biomass is also negatively affected (Saeidi et al., 2015; Ding et al., 2018). This results in changing source-sink relationships, resulting from an increased fraction of available carbon being allocated to the root system rather than to the shoot when plants are under limited water supply (Palta and Gregory, 1997).

Contrary to the above observations, an improvement in canopy structure and also maintenance of photosynthesis at the canopy level was observed when mild water stress was applied at stem elongation without a reduction in yield (Liu et al., 2016; Table 1). It is sometimes argued that mild drought stress during this phase may not be very critical to the final grain yield. One possible explanation for these latter observations is that mild drought stress during tillering and stem elongation stages primes wheat plants to become acclimated to tolerate drought during the grain filling period (Wang et al., 2015); the mechanism involves low accumulation of hydrogen peroxide (H_2_O_2_) due to increased activity of H_2_O_2_ scavenging enzymes such as ascorbate peroxidase (APX) and guaiacol peroxidase (POX) (Khanna-Chopra and Selote, 2007). Despite some reports (Wang et al., 2015; Liu et al., 2016), the above evidence suggests that drought at tillering and stem elongation stages negatively affects grain yield. Therefore, the evaluation of genotypes for drought tolerance at these growth stages is equally important as other growth stages.

### Heading and Anthesis Stages

Many studies suggest that flowering and anthesis are the most susceptible wheat growth stages to drought (e.g., Ji et al., 2010; Fahad et al., 2017; Sarto et al., 2017). Water stress at heading and anthesis stages causes multiple impacts, but amongst these, a decrease in the number of grains per head and grain weight was reported to be the most severe (Varga et al., 2015; Table 1). Drought at these stages reduces pollen viability leading to failures in fertilization, and thus spikelet sterility (Ji et al., 2010; Su et al., 2013). This is also the stage when there is maximum evapotranspiration, which aggravates the situation and leads to severe crop loss. On the other hand, in comparison to severe drought, moderate stress at these stages may improve the translocation of assimilates in some genotypes (Liu et al., 2016; Ding et al., 2018) but this observation may be specific to only a few genotypes.

### Grain Filling Stage

It might be expected that drought during grain fill would be extremely critical compared to earlier growth stages because there is less opportunity for recovery compared to earlier stages. However, various studies (Table 1 and noted below) suggest that this stage is not more sensitive to drought, suggestive of mitigation strategies. Indeed, at the grain filling stage, though water availability becomes critical for translocating photosynthates to the grain, pre-anthesis storage reserves such as those in the stem can play vital roles in preventing yield loss, to mitigate the negative impact of moisture stress on photosynthate assimilation (Blum, 1998; Liu et al., 2016). One study showed a 5.2% decrease in kernel weight and a 20.7% reduction in kernel numbers as a result of drought imposed after anthesis resulting in a ∼28% yield decline (Gevrek and Atasoy, 2012; Table 1), indicating the severe impact of drought during the grain filling period.

Furthermore, mild drought during the grain filling stage does not appear to cause a significant reduction in final grain yield (Ding et al., 2018). As noted above, moderate drought during vegetative growth stages may prime plants to acclimate to drought during grain fill; the mechanism involves reduced photo-inhibition in the flag leaves at this later stage associated with increased accumulation of abscisic acid (ABA) (Wang et al., 2015). In addition, drought tolerance during grain fill may be due to the accumulation of dehydrins (Lopez et al., 2002), a family of hydrophilic, thermostable proteins produced during dehydration that provide protection from a yet unknown mechanism (Yu Z. et al., 2018).

## Phenotyping Above Ground Physio-Morphological Traits Associated With Drought Tolerance at Different Growth Stages

**FIGURE 4:**
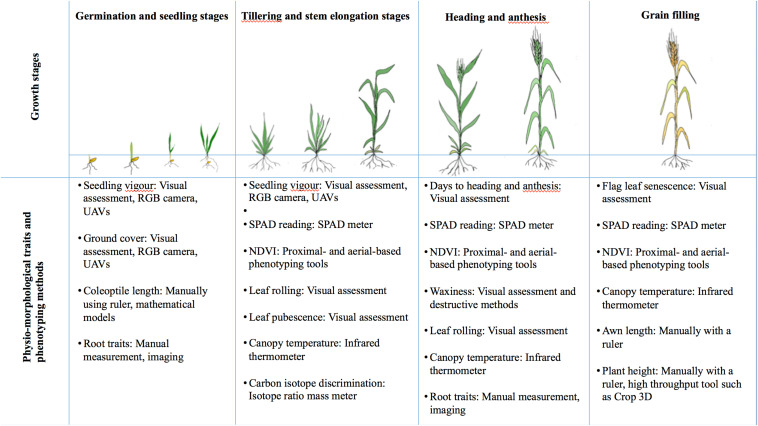
Summary of physio-morphological traits associated with different growth stages and phenotyping methods in wheat.

In the previous section, the impact of drought during specific growth stages of wheat was described. For these observations to be used in a breeding program, it is important to identify phenotypic target traits for selection at each of the growth stages. Phenotypic-based selection is already a common practice in breeding for drought-tolerant wheat varieties. To further improve the efficiency of breeding, researchers have identified many more useful physio-morphological traits that influence drought tolerance, and these candidate traits for selection are summarized in Figure 4 and detailed below.

### Germination and Seedling Growth Stages

#### Coleoptile Length and Gibberellic Acid (GA_3_) Sensitivity

The coleoptile of wheat is a pointed protective sheath, which covers the emerging shoot or the first leaf during germination (Farhad et al., 2014). When a seed germinates, the coleoptile pushes through the soil and protects the young shoot. Coleoptile length is measured from the scutellum to the tip of the coleoptile sheath (Rebetzke et al., 2005) when the coleoptile is completely visible, typically within 1–2 weeks after germination. The length of the coleoptile varies among different genotypes, and this has special importance to wheat cultivation in drought environments. The concept of deep planting is more subjective as the depth seeding may be defined based on the soil moisture availability. Although coleoptile length may not be the sole factor responsible for emergence after deep sowing (Mohan et al., 2013), the frequency of emergence under these conditions, when combined with the threat of surface desiccation, is greater in genotypes with longer coleoptiles than those with shorter coleoptiles (Rebetzke et al., 2005, 2007; Farhad et al., 2014). Poor plant establishment can, therefore, result from shorter coleoptiles under stressed conditions (Schillinger et al., 1998), especially in dry environments. All of these studies establish that there is potential to use coleoptile length as a surrogate trait to screen drought-tolerant wheat varieties. However it is not appropriate to select for coleoptile length solely based on its impact on early-season drought tolerance. Nevertheless, the above evidence suggest that it is a trait that carries potential while breeding wheat for early-season drought tolerance.

It is noteworthy that the dwarfing genes in wheat have important associations with coleoptile length. Specifically, over the past few decades of modern wheat breeding, the dwarfing genes *Rht-B1b* (*Rht1*) and *Rht-D1b* (*Rht2*) derived from “Norin-10,” a Japanese wheat variety, have been widely used to develop dwarf and semi-dwarf wheat varieties around the world (Borojevic and Borojevic, 2005; Waddington et al., 2010). The *Rht1* and *Rht2* genes made the plants insensitive to internal gibberellic acid (Keyes et al., 1989) leading to shorter coleoptiles and reduced plant height as a result of reduced cell elongation (Rebetzke et al., 2001). There are other dwarfing genes apart from *Rht-B1* and *Rht*-*D1* that are sensitive to endogenous gibberellins, which reduce plant height but have less or no effect on coleoptile length and leaf surface area (Rebetzke et al., 1999, 2005; Ellis et al., 2005). The dwarfing gene *Rht8*, derived from the Japanese variety “Aka Komugi” (Grover et al., 2018), allows for the development of a long coleoptile (Rebetzke et al., 2005). The *Rht8* gene has been employed in commercial varieties in some Mediterranean countries including Italy. According to Grover et al. (2018), coleoptile length and plant height were reduced by only 6.75 and 2.84%, respectively, in the lines carrying the *Rht8* gene compared to 21.64 and 23.35% reductions in lines containing the *Rht1* gene.

Growing seeds on filter paper or germination paper under dark conditions (Purchase et al., 1992) is a common practice while the “cigar-roll method” (Bai et al., 2013) is another method more into use. The coleoptile measurements are also taken after growing seeds in soil media (Mohan et al., 2013). Measurement of coleoptile length is commonly done manually when the seedlings are 7–10 days old, and it is a time taking activity. Recently, Zhang C. et al. (2018) analyzed coleoptile images using custom-developed image processing algorithms which were significantly correlated (*r* = 0.69–0.91, *p* < 0.0001) with manual measurement of coleoptile length.

#### Seedling Vigor

Among phenotypic targets for selection, seedling vigor is considered as a candidate trait for evaluating wheat for drought tolerance at the early growth stage (Rebetzke et al., 1999; Reynolds et al., 2005). The four-leaf stage is the appropriate stage to study seedling vigor (Hafid et al., 1998). This trait is characterized by the extent of ground cover and establishment, and can be measured using a visual rating score (Mullan and Garcia, 2012). Also, the use of digital imaging techniques such as using an RGB camera is also a common way to assess seedling vigor, though this requires technical skills to process the images using different software programs. One of the reasons why this is a promising trait is the large number of genotypes that can be screened quickly and cost effectively (Dhanda et al., 2004). Dodig et al. (2015) found a significant association (*r* = 0.31, *p* ≤ 0.01) between a stress tolerance index (STI) at the seedling stage and the STI associated with grain yield in wheat. Furthermore, early seedling vigor has been reported to have a positive correlation (*r* = 0.28–0.63, *p* < 0.01) with grain yield (Kandic et al., 2009). Vigorous, healthy seedlings are water and nutrient use efficient and can compete against weeds (Zhang et al., 2014). A handful of scientists have recently emphasized the use of seedling vigor as part of variety selection: for example, Ahmad et al. (2015) evaluated 50 wheat genotypes for different seedling traits including seedling vigor and successfully identified eight potentially drought-tolerant genotypes. Such evidence highlights the importance of this trait in breeding wheat for drought tolerance. Recent studies (Zhang C. et al., 2018; Walter et al., 2019) showed that digital imaging using an RGB camera that recognizes color-based traits may be an alternative to visual assessment which is more subjective. These studies have highlighted that digital imaging may increase the throughput rate and precision of measuring seedling traits. The images can be analyzed by using open source software such as ImageJ (Schillinger et al., 2010). Therefore, application of high throughput imaging technologies can create opportunities to more efficiently use this trait for screening in breeding programs.

### Vegetative Growth to Post-anthesis Growth Stages

#### Number of Tillers

Different studies have reported that drought reduces the number of tillers (Maqbool et al., 2015; Abid et al., 2018). Since each tiller has the potential to initiate a spike, then the tiller number directly affects grain yield in wheat. Tiller initiation in wheat is asynchronous, and the tillering stage encompasses newly initiating tillers as well as older tillers undergoing reproductive transition. Therefore, the tillering stage determines both the tiller number and also the development of reproductive primordia (spike, spikelets, and florets) (Figure 3). Indeed, moisture stress during this stage reduces tiller number which is compounded by a reduction in the number of kernels per spike (Blum et al., 1990). The extent to which moisture stress reduces tiller number varies among genotypes depending on the intensity and duration of stress (Sarto et al., 2017). The genotypes that maintain a higher rate of photosynthesis and other physiological parameters such as leaf water potential, and exhibit rapid recovery after re-supplying moisture, promote drought tolerance at the tillering stage (Abid et al., 2018). In many cases, the number of tillers may not be reduced significantly if the moisture is supplied before the end of the tillering stage, but in such cases, the tillers have a low biomass and kernel weight (Blum et al., 1990). There are also arguments that the number of tillers may not always be a good indicator of yield in wheat. Duggan et al. (2000) showed that under drought, vigorous tillers each with a high number of large kernels were more important than the numbers of tillers *per se* in terms of grain yield. Although many researchers do not measure tiller number, it is an important trait that should be considered in breeding for drought tolerance in wheat. This trait is commonly scored at maturity by counting the number of tillers per unit area in a representative part of each plot.

#### Chlorophyll Content

The green area of plant leaves specifies a plant’s photosynthetic capacity and provides valuable information associated with crop productivity, and the physiological and phenological status of the plant (Kira et al., 2015; Ramya et al., 2016). Chlorophyll content positively correlates with grain yield (Talebi, 2011; Kumari et al., 2012). Abiotic stresses lead to changes in the amount of chlorophyll content in plant leaves. Drought susceptible wheat varieties showed losses in chlorophyll content, while tolerant varieties exhibited higher chlorophyll content (Khayatnezhad et al., 2011). Similarly, a 13–15% reduction in chlorophyll content was observed in wheat varieties due to limited water supply (Nikolaeva et al., 2010). This loss in chlorophyll content is due to decreased expression of genes encoding enzymes for chlorophyll biosynthesis (Liu et al., 2018). By contrast, the stay-green trait in which chlorophyll persists, and which signifies the delayed senescence of leaves, is another important indicator of stress tolerance (Rosyara et al., 2009; Lopes et al., 2012) and is discussed in more detail below. Talukder et al. (2014) state that stress during anthesis results in a significant drop in flag leaf chlorophyll content and yield-related traits such as grain number, grain mass and duration of grain filling. Khayatnezhad et al. (2011) observed that the durum wheat varieties that had a significantly higher chlorophyll content during the reproductive phase were higher yielding and more tolerant to end-season drought. Other studies have also revealed that drought-tolerant wheat genotypes maintain higher chlorophyll content during water stress compared to drought susceptible varieties (Talebi, 2011; Bowne et al., 2012; Rehman et al., 2016; Ahmed et al., 2019). All of these examples delineate that crop varieties with higher chlorophyll content and slow chlorophyll degradation, are potentially more drought-tolerant. Therefore, chlorophyll content is an important trait that can be used as a proxy for drought tolerance and higher grain yield, and it is a simple trait to phenotype.

The Soil Plant Analysis and Development (SPAD) meter can rapidly measure SPAD values as a proxy for chlorophyll content (Rosyara et al., 2009; Lopes et al., 2012) and is widely used. The SPAD meter has diodes that emit red and near infra-red wavelengths that pass through the leaf; chlorophyll absorbance is determined at 650 nm while non-chlorophyll absorbance is calculated using a wavelength peaking at 940 nm (Lopes et al., 2012). In wheat, SPAD measurements are taken from the flag leaf when evaluating varieties for selection (Paul et al., 2016). SPAD measurements can be taken starting at the emergence of flag leaves until early senescence at a desired time interval (e.g., a week or 10 days). This allows a breeder to assess the association of chlorophyll content at different growth stages with other target traits such as grain yield (Rosyara et al., 2009).

#### Normalized Difference Vegetative Index

The normalized difference vegetative index (NDVI) is a metric for vegetation based on the difference between the reflection of near-infrared light (which vegetation strongly reflects) and red light (which vegetation strongly absorbs) (Sultana et al., 2014). Values of NDVI range from −1 to +1, and are calculated using the following formula:

$$N\,D\,V\,I=\frac{R_{N\,I\,R}-R_{R}}{R_{N\,I\,R}-R_{R}}$$

Where:

NDVI = Normalized difference vegetative index;

R_*NIR*_ = Near-infrared radiation;

R_*R*_ = Visible red spectrum.

NDVI is extensively used as a trait to report the greenness and ground cover of the vegetation, and therefore, to infer the photosynthetic strength of the crop canopy, using measurements taken from the ground level up to satellite altitudes (Pietragalla and Vega, 2012). It is now widely accepted as a proxy for drought adaptive traits (Lopes and Reynolds, 2012), having a positive association with grain yield (Ramya et al., 2016; Condorelli et al., 2018), and it has also shown potential for estimating growth rate, seedling vigor and senescence patterns in wheat (Pietragalla and Vega, 2012). For example, selection for yield parameters based on NDVI along with other chlorophyll measurements resulted in a yield increase by 17.1% in a half-sib population of bread wheat (Ramya et al., 2016).

The GreenSeeker spectral sensor (Trimble, CA, United States) is an NDVI tool that has been used to measure the greenness of a plant and is commonly used to estimate the greenness of an entire field plot. The use of GreenSeeker has been considered more integrative than SPAD as it is mostly helpful in determining the pattern of senescence from the whole crop canopy, while SPAD uses only one leaf at a time to record the greenness. However, GreenSeeker is unique as it only measures the reflectance of the modulated red and NIR light that it provides, not the ambient light. The accuracy of the measurement depends upon the number of NDVI measurements taken (Lopes et al., 2012). Therefore, recording NDVI throughout the crop season at defined intervals is essential to improve the accuracy of the measurements taken and to evaluate the pattern of change in greenness throughout the season. Although NDVI is a commonly used index, its sensitivity to genetic and environmental diversity has been criticized (Christopher et al., 2016). Therefore, caution should be used when using NDVI to predict wheat grain yield when a diverse genetic population is involved and when the environmental conditions are highly variable. While NDVI can be the first choice for many breeders, the use of other vegetative indices such as Normalized Difference Red Edge (NDRE) may be another option. The performance of NDRE is better compared to NDVI, overcoming the limitations of NDVI associated with absorptance by the upper canopy and saturation at its maximum value during later growth stages of the crop (Fu et al., 2020).

#### Chlorophyll Fluorescence

Chlorophyll fluorescence measurements can be used to non-destructively determine a wide variety of leaf-level parameters related to the functional status of Photosystem II (PSII), the first protein complex in the light-dependent reactions of photosynthesis. For example, in illuminated leaves, chlorophyll fluorometry can be used to calculate the rate of photosynthetic electron transport through PSII (Genty et al., 1989; Earl and Tollenaar, 1998), or to quantify the activity of non-photochemical quenching processes associated with the safe dissipation of excess light energy (Laisk et al., 1997).

More commonly, chlorophyll fluorescence is measured on dark-adapted leaves (e.g., pre-dawn, or after placing plants in darkness for a defined period, generally 30 min to a few hours). Dark-adapted measurements provide estimates of maximum efficiency of PSII, which can be reduced due to damage or down-regulation occurring in response to prior stress (Lu et al., 2003; Baker, 2008). Because PSII efficiency is so sensitive to any stress affecting photosynthesis, evaluation of chlorophyll fluorescence can be used as a quick indicator in any crop (Jansen et al., 2014). The parameters of chlorophyll fluorescence measured on dark-adapted leaves, namely the initial fluorescence (*F*_0_; the signal when all functional PSII centers are “open”), maximum fluorescence (*F*_M;_ the signal when all PSII centers are “closed” by a brief pulse of saturating light), variable fluorescence (*F*_V_ = *F*_M_ – *F*_0_) and efficiency potential (*F*_V_/*F*_M_) are affected by unfavorable environmental factors like drought (Zhang et al., 2010; Sharma et al., 2014a). Under drought conditions, *F*_0_ may increase or decrease, while *F*_M_, *F*_V_ and *F*_V_/*F*_M_ all decrease. These parameters of chlorophyll fluorescence kinetics (PCFKs) and imaging are considered as very powerful and reliable indicators of the impact of various abiotic stresses, including drought, on plant physiological processes (Paknejad et al., 2007; Khayatnezhad et al., 2011; Jansen et al., 2014; Sharma et al., 2014b). Under drought conditions, crop varieties that maintain high *F*_V_/*F*_M_ under water-limited conditions are considered to be stress tolerant (Zlatev, 2009), and indicate efficient protection of PSII activity. Chlorophyll fluorescence has been used in a diversity of plant species including wheat (Zlatev, 2009; Khayatnezhad et al., 2011; Rahbarian et al., 2011; Kamanga et al., 2018; Yao et al., 2018). Similarly, analysis of PCFKs in winter wheat seedlings indicated that the variety that maintained *F*_V_/*F*_M_ was tolerant to water stress, able to maintain high photosynthetic activity (Zlatev, 2009). Despite the potential usefulness of PCFKs in drought tolerance studies, there are some limitations associated with the measurement of these parameters. In particular, the high-throughput measurement of PCFKs is mostly useful during the seedling stage and can be a difficult task later in development (Furbank and Tester, 2011). Another issue is that these parameters may have limited applicability to breeding programs if the population size is very large, although emerging high throughput phenotyping technologies may overcome this challenge. The best application of PCFKs in breeding may be at the advanced breeding stages where the number of genotypes may not be a limiting factor.

#### Shoot Waxiness

Cuticle, the outermost layer of the plant shoot, is made up of cutin and waxes. The amount of wax present on the leaves differs among species and genotypes within a species. Waxiness, also known as glaucousness, is a shoot morphological trait that is vital from the perspective of environmental stress tolerance (Bi et al., 2017). In wheat breeding, waxiness is used to select drought-tolerant genotypes. The composition of the wax and the changes in wax composition influence water loss from the cuticular surface, leading to drought tolerance, though it is not the sole indicator (Jäger et al., 2014; Bi et al., 2017). In a study consisting of three wheat varieties, Bowne et al. (2012) reported that the variety with the most waxiness yielded the highest under both mild and severe drought conditions, supporting that waxiness reduces the loss of water from plant surfaces. Waxiness is usually quantified visually based on the proportion of visible bluish-white colored wax on the plant shoot surface including spikes when phenotyping in the field. A 0–10 visual rating scale is commonly used, where 0 indicates no or low waxiness, and 10 indicates high waxiness (Torres and Pietragalla, 2012). Qualitatively, analyses of cuticular waxes have shown that genotypes with higher β-diketones, one of the two major components of wax along with alkanes, are more drought-tolerant (Bi et al., 2016, 2017). More recently, it has been shown that considerable changes occur in the composition of carbonyl ester in cuticular wax of wheat leaves when exposed to water stress suggesting the possibility of having a new biochemical marker for drought tolerance (Willick et al., 2018). Considering its influence on drought tolerance, and given that it is easy to score visually, waxiness is a valuable trait to include when breeding wheat for drought tolerance.

#### Carbon Isotope Discrimination

Water use efficiency (WUE), known as transpiration efficiency, is the ratio of above ground (aerial) biomass yield (carbon) to the total amount of water used by a plant (Condon et al., 1992; Rebetzke et al., 2002). Direct measurement of WUE is very complicated and also time exhausting. Therefore, utilization of carbon isotope discrimination (Δ), which is a proxy for the ratio of intercellular (*C*_i_) and the atmospheric (Ca) partial pressure of CO_2_, i.e., *C*_i_/Ca, has been exploited as an indirect measurement of WUE (Farquhar et al., 1982, 1989; Ehdaie et al., 1991). CO_2_ exists in the atmosphere mostly as ^12^CO_2_, but also as the rarer, heavier isotope ^13^CO_2_. During CO_2_ fixation, plants discriminate between these two isotopes, favoring ^12^C since ^12^CO_2_ diffuses more freely into leaves than does the heavier isotope, and also because RuBisCO fixes ^12^CO_2_ more readily than ^13^CO_2_. However, the magnitude of discrimination (and therefore the measurable ^13^C/^12^C ratio in the final crop biomass) varies depending on the type of crop, genotype, environment and other factors (Kumar and Singh, 2009). If C_i_ was in perfect equilibrium with C_a_, then discrimination against ^13^C would be constant. However, because stomatal conductance is not infinite, photosynthesis reduces leaf internal CO_2_ (i.e., C_i_/C_a_ < 1), and the air inside the leaf becomes more depleted in ^12^C relative to ^13^C, which reduces the opportunity for further discrimination in favor of ^12^C. Thus, plants that have had lower C_i_ over their lifetimes will have higher ^13^C/^12^C in their final biomass. Since lower C_i_ is associated with higher WUE (Farquhar et al., 1982), carbon isotope discrimination serves as a time-integrated proxy for WUE. Consistent with this well-established theory, Δ has been shown experimentally to be negatively associated with WUE (Ehdaie et al., 1991; Medrano et al., 2015). The Δ metric has been used to assess WUE and its association with grain yield in many different crops including wheat (Austin et al., 1990; Blum, 2005; Akhter et al., 2008; Kumar and Singh, 2009; Luckett et al., 2011). Interestingly, Δ has high heritability (the narrow-sense heritability is ∼0.63) under limited water conditions (Rebetzke et al., 2002, 2006), and thus, it is a potential indirect method to select high yielding wheat varieties for dry environments (Rebetzke et al., 2006). To determine Δ, finely ground dried shoot tissue is analyzed using an isotope ratio mass spectrometer (Optima, VG Instruments, United Kingdom) (Becker and Schmidhalter, 2017; Bachiri et al., 2018). It has been assessed using the flag leaf in durum wheat under drought conditions by Merah and Monneveux (2001) and in *Triticale* by Munjonji et al. (2016). The throughput rate for determination of Δ may not be high but it is a reliable trait to assess drought tolerance in wheat. The trait may be best evaluated when the wheat breeding lines are at an advanced stage.

#### Leaf Rolling

When plants are under limited water conditions, turgor pressure adjustment in the cells is one of the biochemical mechanisms that helps plants acclimate to dry conditions (Price, 2002; Fang and Xiong, 2015). Leaf rolling is one of the consequences of turgor pressure adjustment observed in diverse plants when they are exposed to limited water environments (Kadioglu and Terzi, 2007; Fleury et al., 2010). During the stress period, leaf rolling reduces the leaf area, which in turn reduces the effective area for evapotranspiration (Fang and Xiong, 2015; Lamaoui et al., 2018) and hence represents a drought acclimation response. In wheat, like other cereals, leaf rolling is a typical symptom when there is water deficit in the soil (Clarke, 1986; Kadioglu et al., 2012). Since leaf rolling is associated with water loss in cultivated wheat, it is a potential proxy trait for screening wheat genotypes up to a certain level of water loss from the leaves (Condorelli et al., 2018). Although the heritability of leaf rolling was found to be high (narrow-sense heritability ∼0.83), Sirault et al. (2008) suggest further investigation to exploit the usefulness of this trait for breeding. In wheat, leaf rolling can be easily scored visually in the field using qualitative scales (Torres and Pietragalla, 2012; Condorelli et al., 2018) at the vegetative and reproductive growth stages. Despite leaf rolling not being widely used, it has the potential to improve the efficiency of breeding for drought tolerance in wheat.

#### Canopy Temperature

Canopy temperature (CT) is a physiological trait that indicates crop water status (Mason and Singh, 2014) and is an established proxy trait for stomatal conductance (Deery et al., 2019). CT has the potential to be a very useful tool for indirect selection of tolerant genotypes for heat and drought stress tolerance (Reynolds et al., 2009b). In such environments, genotypes that maintain cooler canopies are more likely to thrive. It has been observed that in deep-rooted genotypes, CT is usually lower, as the crop can extract moisture from a deeper soil depth (Lopes et al., 2012). The CT is affected by various confounding factors such as solar radiation, soil moisture, wind speed, temperature, and relative humidity (Reynolds et al., 2012; Mason and Singh, 2014), and hence caution should be used when interpreting CT data. CT can be measured from post-tillering to physiological maturity using an infrared thermometer (Pask and Reynolds, 2013), thermal imaging (Costa et al., 2013), and ArduCrop wireless infrared thermometers (Rebetzke et al., 2016b; Deery et al., 2019). Since the frequency of drought and heat stress are expected to increase in the near future, the development of wheat genotypes with cooler canopies should be one of the targets of a wheat breeding program.

#### Days to Heading and Anthesis

According to Singh (1981), there are three very critical phenological stages in wheat, based on the soil moisture requirement: the early vegetative stage, booting to the heading stage, and the flowering and grain filling stage. Many studies suggest that plants are more sensitive to water limitation from heading to flowering than the other stages (Nezhadahmadi et al., 2013; Farooq et al., 2014; Sarto et al., 2017). Evapotranspiration reaches a maximum at this growth phase (Sarto et al., 2017). Therefore, maintenance of optimum soil moisture at this interval is critical to ensure higher wheat yield and yield stability (Senapati et al., 2019). Drought at heading has been found to affect spike weight negatively (Ding et al., 2018). Similarly, in winter wheat, Varga et al. (2015) reported that water stress during the heading period, or the growth stages after heading, resulted in a higher yield penalty. Drought at the onset of flowering results in sterility and a reduced number of grains per spike, which is primarily due to reduced pollen viability and ovule abortion as a consequence of limited moisture (Su et al., 2013). With respect to genetic variation in this trait, in a study that evaluated the response to drought during the reproductive stage, Senapati et al. (2019) observed a 13.4% higher mean yield in drought-tolerant wheat genotypes compared to susceptible genotypes across 13 major wheat growing regions of Europe. One additional study showed that early heading and anthesis can be escape mechanisms for terminal drought (Shavrukov et al., 2017), perhaps more applicable to geographic regions that suffer from late-season water deficit. Phenotyping a genotypically diverse set of plants at heading and anthesis stages is time-consuming, requiring daily visits to a field site, because of the corresponding diversity in flowering times. Recently, an automated phenotyping method for these stages has been tested in wheat, involving computer software analyzing digital images; the method has achieved a >85% accuracy for both stages (Sadeghi-Tehran et al., 2017). Based on its reproducibility and cost effectiveness, such a method has potential for adoption in wheat breeding programs.

#### Flag Leaf Senescence

It is proposed that a delay in flag leaf senescence greatly influences grain yield in cereal crops (Thomas and Howarth, 2000; Borrill et al., 2015). In wheat, early flag leaf senescence has been found to affect grain yield negatively (Gregersen et al., 2013) including under dryland conditions (Liang et al., 2018), while delayed flag leaf senescence is positively associated with higher grain yield (Verma et al., 2004) and harvest index (Carmo-Silva et al., 2017). Drought-tolerant varieties have a higher CO_2_ uptake rate in the flag leaves which contributes to yield stability (Paul et al., 2016). During the senescence phase, protein complexes in chloroplasts including PSII, break down which leads to changes in the structure, metabolism and gene expression of photosynthetic cells, ultimately resulting in loss of cellular chlorophyll content (Thomas and Howarth, 2000; Podzimska-Sroka et al., 2015). Therefore, to maintain translocation of assimilates from leaves and stems to the grain and maintain grain yield, genotypes with delayed leaf senescence are preferred (Hafsi et al., 2013). Delayed senescence and persistence of greenness were introduced above as the stay-green trait and are associated with altered cytokinin and ethylene activities (Thomas and Ougham, 2014). This evidence implies that delayed flag leaf senescence can be a key parameter for selecting genotypes in areas with end season drought stress (Campos et al., 2004). Flag leaf senescence can be recorded visually by using qualitative leaf color scores (Pask and Pietragalla, 2012). There are some studies with wheat which reveal that the contribution of ear photosynthesis to grain yield is relatively greater than that of the flag leaf in optimal and drought environments (Abbad et al., 2004; Bragado et al., 2010; Yun-qi et al., 2016). Nonetheless, the significance of delayed flag leaf senescence for drought tolerance should not be underestimated. Since flag leaf senescence is easy to record and has close association with chlorophyll decline, the trait can be targeted in wheat breeding programs for drought tolerance. Confounding effects may be observed, however, when the genotypes under study are extremely diverse with respect to days to maturity.

#### Grain Filling Rate and Duration

In cereals, the grain filling period is one of the most sensitive growth stages to water stress (Barnabás et al., 2008; Alghabari and Ihsan, 2018) and commonly affects many wheat growing regions of the world including semi-arid regions (Saeidi and Abdoli, 2015). Grain yield loss depends on the severity of the stress during this interval (Farooq et al., 2014; Tabassam et al., 2014; Saeidi and Abdoli, 2015). This loss in grain yield could be due to a reduction in the duration of grain filling (Alghabari and Ihsan, 2018) which is associated with early senescence (Farooq et al., 2014; Bogale and Tesfaye, 2016). Monpara (2011) stated that the grain filling period is positively correlated (*r* = 0.51, *p* < 0.05) with grain yield in wheat, which was confirmed under water-limited condition (Kilic and Yagbasanlar, 2010; Bogale and Tesfaye, 2016). Ihsan et al. (2016) observed that a drought-tolerant wheat genotype had a 38% longer grain filling period compared to a drought susceptible genotype. Other researchers such as Nass and Reiser (1975) and Borrill et al. (2015) argue that the rate of grain filling or grain filling capacity is more critical than the grain filling duration. Madani et al. (2010) and Baillot et al. (2018) showed that the rate of translocation of photosynthates to the grain contributes to grain weight and is a major component of grain yield in wheat, which supports the statement that grain filling rate is more fundamental to grain yield than grain filling duration. There is also an argument that stressed plants may have a higher rate of grain filling, and this, combined with a shorter grain filling period, cause improper assimilate translocation which ultimately leads to reduced grain yield (Ihsan et al., 2016; Alghabari and Ihsan, 2018). Therefore, it may be useful to screen genotypes that have a longer interval from anthesis to grain maturity and/or a higher rate of grain biomass increase in a time course. Selection for a higher rate of grain biomass increase may be more useful than delayed grain maturity in climates where short duration wheat varieties are grown.

#### Awn Length

Wheat genotypes vary for having spikes with no awns to those with differing awn lengths. Awn length is measured from the tip of the spike to the tip of the longest awn but can also be scored qualitatively (Torres and Pietragalla, 2012). A significant positive association was observed between awn length and grain yield, and between awn length and spike length, under water deficit conditions (Blum, 2005; Taheri et al., 2011; Khamssi and Najaphy, 2012; Ali et al., 2015). A significant correlation (*r* = 0.43, *p* ≤ 0.01) between awn length with drought stress tolerance index was also observed (Taheri et al., 2011). Similarly, it was observed that awns maintained a higher relative water content and photosynthetic electron transport rate compared to the flag leaf under drought, indicating tolerance to soil moisture deficit (Maydup et al., 2014). Awns are green and contribute to the photosynthetic area, and positively influence grain yield (Rebetzke et al., 2016a). Maydup et al. (2010) reported that the contribution to grain yield by spike photosynthesis was up to 42% while the contribution was even greater in varieties having longer awns. The results from another study (Towfiq and Noori, 2016) showed that the awns increased the photosynthetic spike surface area by 36–59% and increased grain yield by 10–16%. Morphologically, moisture stress causes a significant reduction in awn dry weight but not length (Khamssi and Najaphy, 2012). This result may be related to awns competing for assimilates during ovary growth, and represents another issue that requires further investigation. Furthermore, awns are associated with larger but fewer grains, which is another topic that needs to be further investigated (Rebetzke et al., 2016a). Integrating the information discussed above (Taheri et al., 2011; Ali et al., 2015) and its high to moderate level of heritability (broad-sense heritability ∼0.95) (Bhatta et al., 2018), awn length is an interesting trait to consider for assessment of wheat genotypes for drought tolerance. Awn length is measured from the tip of the spike to the tip of the longest awn after physiological maturity (Torres and Pietragalla, 2012).

#### Plant Height

Plant height is one of the critical traits affected by drought in wheat. Low moisture reduces photosynthesis and metabolite/nutrient translocation in wheat, especially during the stem elongation stage, resulting in reduced height (Sarto et al., 2017). The reduction in plant height is due to altered carbon partitioning as the plants undergo osmotic adjustment under moisture stress (Blum and Sullivan, 1997). The proportion of reduction in plant height depends on both the intensity of drought and the genotype. Reductions in wheat plant height of 2–24% and 1–16%, respectively, due to drought stress applied at 30 and 70% field capacity during the stem elongation stage, have been reported (Qadir et al., 2016). Similarly, in another study (Mirbahar et al., 2009), a ∼25% reduction in wheat plant height was observed due to drought. The drought tolerant plants tend to maintain shorter plant height and plant area index to reduce the moisture demand and prevent moisture loss due to transpiration (Su et al., 2019). Therefore, proportionally, the growth reduction observed in smaller plants is less than larger plants indicating that smaller plants are less sensitive to moisture stress (Blum and Sullivan, 1997). With respect to this concept, selection for a higher root to shoot ratio appears to be a more appropriate strategy for drought-prone environments (e.g., Ahmed et al., 2019). Plant height in wheat is commonly measured manually using a ruler after maturity. However, sensor-based tools have been recently tested in other crops for the measurement of plant canopy height (Andrade-Sanchez et al., 2014) which can be transferred to wheat breeding programs to reduce time and labor.

## Phenotyping Root Architectural Traits

Aside from the above-ground traits described above, root system architecture (RSA) also plays a vital role in the growth, development and overall productivity of crop plants (Wasaya et al., 2018). In general, RSA of a plant largely depends upon the environment within which it grows and the types of abiotic stresses associated with soil and water that it experiences (Wang and Frei, 2011). When water is the major constraint, then root characters have an equally important role as shoot traits in promoting drought tolerance (Manavalan et al., 2010). Some of the important root traits, such as root angle, primary root length (Comas et al., 2013; Forde, 2014), the number of lateral roots (Zhan and Lynch, 2015), and average root diameter (Comas et al., 2013; Haling et al., 2013), contribute to regulating water uptake which is critical under drought stress. It is stated that plants with root systems having a smaller root diameter and long fine roots are more suited to drought environments.

In wheat, the RSA consists of two different types of roots, the early initiating seminal roots, sometimes regarded as embryonic roots, and the later nodal roots, commonly known as a crown or adventitious roots (Manske and Vlek, 2002; Kirby, 2002). The wheat embryo develops ∼6 root primordia: during germination, the primary root and 4–5 seminal roots emerge out of the coleorhiza which ultimately develops into the seminal root system (Perry and Belford, 2000; Kirby, 2002). The later nodal roots, which emerge at the nodes, develop as tillering progresses (Kirby, 2002; Manske and Vlek, 2002). The seminal roots can grow up to 2 m deep into the soil, while the nodal roots are thicker and scavenge from the upper surface of the soil in a more horizontal orientation, to the middle layers (Kirby, 2002). Both seminal and nodal roots branch to form lateral roots.

Similar to other crops, root traits are also associated with drought tolerance in wheat (Lopes and Reynolds, 2010). Root angle is a trait that explains some mechanisms related to soil-root interactions (Chen et al., 2017, 2018). The seminal and nodal roots of genotypes with a narrow root angle tend to grow deeper compared to those with a wider root angle at the early growth stages (Manschadi et al., 2008; Wasson et al., 2012; Richard et al., 2015). Similarly, a high density of lateral roots at a narrow angle (steeper) are considered better for wheat since such roots have more access to soil moisture at a deeper soil depth (Manschadi et al., 2008; Wasson et al., 2012; Chen et al., 2017, 2018). Root angle in wheat shows high heritability (broad-sense heritability ∼0.82), and thus, it is an important trait to consider when breeding wheat for drought tolerance (Hassouni et al., 2018). Although evidence suggests that root angle is a vital trait especially for end season drought tolerance in wheat (Gesimba et al., 2004), it is important to note that wheat root systems that have a high number of nodal and seminal roots in the crown region located close to the surface of the soil are more drought-tolerant at the early growth stages.

The limited number of studies on RSA morphology is primarily due to a lack of efficient high throughput phenotyping methods. Conventional methods of root study include excavation of roots from the field which is labor intensive and time-consuming; such methods are mostly limited to phenotyping young roots under controlled conditions. For example, Goron et al. (2015) established a growth system consisting of fertigation of coarse Turface clay, rather than fine sand or soil which damages roots upon excavation, to phenotype root hairs in wheat, millet, and maize. The study demonstrated that this approach could be an alternative method to characterize finer root traits like root hair number and length. However, as stated above, this method requires considerable time, human and financial resources, which limits its application in plant breeding. Some low-cost and simplified high throughput phenotyping methods have been developed recently. Richard et al. (2015) used two different methods, the clear pot method, and the transparent growth pouch method, to study wheat seedling RSA using two proxy traits, root angle and the number of seminal roots associated with root surface area. In the clear pot method, the pots are transparent, and seeds are sown at the edge of the pots to permit RSA observation; the pots are placed in dark pots to prevent light exposure. Hassouni et al. (2018) also used the clear pot method to study the seminal roots of durum wheat. The use of root digital imaging techniques has also facilitated RSA studies in recent years. Platforms that are capable of automatic washing of the roots are available, as well as machines that can scan the root samples and different software that can analyze the root scanned images (Lobet et al., 2011). Digital imaging of root traits (DIRT) (Bucksch et al., 2014) is an example of such methods, which is useful in characterizing the roots of both dicot and monocot crop species. The use of WinRhizo Tron MF software (Regent Instrument Inc., Quebec, Canada) is another method that has been used to analyze seedling root traits. Tomar et al. (2016) used WinRhizo software to study wheat seedling root traits targeting drought tolerance which included root length, surface area of root, and root volume.

Recently other rapid methods have been developed such as X-ray tomography that provide 3-D images of root systems, which shows significant potential for studying RSA without disturbing the root system (Wasaya et al., 2018). Furthermore, structural root models are also coming into use to analyze RSA traits while model generated images are used to improve the precision of root trait estimation (Lobet et al., 2017). Studies of root foraging, the process by which RSA adapts to changes in the environment, is also another area that is gaining attention (Tian and Doerner, 2013; Chen et al., 2018).

In general, phenotyping mature roots is much more complex and demands more labor than juvenile roots, which limits selection strategies. Selection based on phenotyping seedling roots in a controlled environment may be unreliable, as the traits may not translate into mature RSA under field conditions (Wasson et al., 2012) and hence should be evaluated on a case by case basis (Comas et al., 2013). A method for nodal root phenotyping often referred to as “shovelomics” (Trachsel et al., 2011), where roots are excavated from the soil as already noted above, is in wide use for field phenotyping of root systems, including wheat (Kadioglu and Terzi, 2007). Thus, despite the above mentioned advancements in root phenotyping, the challenge that persists is the lack of cost-effective, high resolution, efficient, simple, and reproducible high-throughput methods that can be readily employed in plant breeding programs (Chen et al., 2018).

## Improving the Efficiency of Phenotyping Physio-Morphological Traits

To make use of a wide range of physio-morphological traits of interest while selecting drought-tolerant wheat varieties, numerous drought screening methods have been developed and tested for use in crop breeding programs (Siddig et al., 2013; Gómez-Luciano et al., 2014; Zu et al., 2017). In the following sections, we review some of the advancements that have been made to improve phenotyping.

### Application of Controlled Physical Structures

One of the greatest challenges in breeding crops for drought tolerance is the simulation of drought at a larger scale. Conducting drought experiments in open field conditions requires a long-term data set as there is a large variation in precipitation and soil moisture conditions (Kant et al., 2017). Additional environmental factors like light intensity, temperature, and canopy cover are also critical in open field experiments, but these factors are highly variable temporally and spatially. Therefore, multi-season, multi-treatment experiments, which enable the understanding of the complex nature of drought, are required (Hoover et al., 2018). Physical structures that overcome the unpredictability of weather while attempting to mimic actual field environments have already shown great potential in drought studies. For example, rainout/rainwater shelters, which are structures established in the field to protect against rainfall, can be used to conduct controlled water experiments over a short span of time (Yahdjian and Sala, 2002; Trillo and Fernández, 2005; Mwadzingeni et al., 2016; Kundel et al., 2018). Rainwater shelters have been successfully tested in different crops including wheat (Zu et al., 2017; Kundel et al., 2018; Wimmerová et al., 2018). Rainwater shelters can be mobile (Wimmerová et al., 2018) or geographically fixed (Kundel et al., 2018) and either manual, semi-automated or fully automated (Ries and Zachmeier, 1985). Custom-designed, automated and portable shelters are very efficient for stress tolerance phenotyping (Kant et al., 2017). For improving the precision of these shelters, it is important that they are equipped with standard soil moisture sensors, motion cameras, and other tools which essentially add value to precise data recording (Mwadzingeni et al., 2016; Kant et al., 2017). One technical issue is that rainwater shelters exclude solar radiation (Wimmerová et al., 2018). Therefore, Vogel et al. (2013) suggested the use of control treatments to assess any confounding effects of these closed structures. The use of these artificial structures for drought tolerance studies is still scrutinized as they do not entirely reflect the real field environment, and very high investments are required to maintain and manage these structures. Despite these limitations, rainout shelters continue to hold promise in efforts to breed for drought tolerance in wheat. This method appears to be the best option available for the study of drought tolerance under field conditions.

### High-Throughput Phenotyping Platforms (HTPPs)

With recent advances in genomics, efficient phenotyping has become limiting (Salas Fernandez et al., 2017). Although it has been common practice to conduct high throughput phenotyping under environmentally controlled conditions (greenhouses, growth rooms, and growth chambers) using single plant measurements, translation of the quantitative trait loci (QTLs) identified under such conditions to the final field-level performance of the crop has been low (White et al., 2012). Since the actual field environment is highly heterogeneous, canopy level phenotypic measurements are considered more accurate. In such a situation, utilization of HTPPs are needed that are precise, labor-, and cost-effective, to phenotype complex physio-morphological traits associated with biotic and abiotic stresses (Rutkoski et al., 2016) as they bridge the gap between genomics and phenomics (Araus and Cairns, 2014; Fahlgren et al., 2015; Bai et al., 2016; Zhang et al., 2017). Innovations in remote sensing, aeronautics and computing have largely contributed to the development of several phenotyping platforms including both ground-based and aerial systems (White et al., 2012; Araus and Cairns, 2014). However, highly sophisticated HTPPs with fully automated functions for canopy level studies are available only in a limited but growing number of institutions such as the United States Department of Agriculture (USDA), the Australian Plant Phenomics Facility and European Plant Phenotyping Network (Araus and Cairns, 2014). Thus, the current situation at many institutions demands judicious selection and proper use of HTPPs that are easily accessible (Cobb et al., 2013; Junker et al., 2015). Management of the high-volume of data generated by these HTPPs also presents a challenge in analysis and interpretation (Shi et al., 2016; Singh et al., 2016; Coppens et al., 2017). In the following two sections, we discuss two high throughput phenotyping approaches that have been gaining attention recently.

#### Proximal High-Throughput Phenotyping Platforms

The use of proximal or ground-level sensors to permit measurements of plant traits at the canopy level has shown potential in terms of replacing current labor-intensive methods. The proximal sensing and imaging approach includes three basic techniques: visible/near-infrared (VIS-NIR) spectroradiometry, infrared thermometry and thermal imaging including conventional digital photography (Araus and Cairns, 2014). These platforms are usually handheld or mounted on vehicles (Qiu et al., 2018). Bai et al. (2016) tested a manually operated multi-sensor system capable of carrying many sensors to measure more than one trait in a wheat breeding program. A strong correlation was observed between grain yield and early and late growth stage sensor-based traits, indicating the significance of using proximal sensing in plant breeding. Similarly, measurement of the vegetative index, NDVI, using a handheld GreenSeeker and a passive bi-directional reflectance sensor, showed similar results in wheat, suggesting that these indices can be used for selection even during early breeding cycles (Walsh et al., 2013; Barmeier and Schmidhalter, 2016). An infrared thermometer has been used to measure CT in wheat (Lopes and Reynolds, 2010), which is very helpful in drought and heat stress studies. Conventional digital cameras have also been used to estimate green biomass, canopy soil cover and plant color (Casadesus et al., 2007; Araus and Cairns, 2014). Some digital cameras with charge-coupled device (CCD) silicon sensors, capable of detecting not only color but also the texture of the objects, are useful in interpreting vegetative indices as well (Qiu et al., 2018). For example, Casadesus et al. (2007) observed a high correlation between vegetation indices from the pictures taken by the digital camera and grain yield in wheat under drought conditions. Comar et al. (2012) developed and successfully tested a semi-automatic system to study the dynamics of change in vegetation index, based on the green fraction (GF), which calculates the fraction of green area per ground area for wheat cultivars grown in micro-plots under field conditions. This system has a hyperspectral radiometer and two RGB cameras that are used to observe the canopy from approximately 1.5 m from the top of the canopy, and it is supported by a tractor that is driven across the plots to collect data from individual plot canopies.

Field-based phenotyping platforms used with other crops may also hold potential for wheat. For example, Andrade-Sanchez et al. (2014) mounted three sensors on a vehicle to measure canopy height, temperature, and reflectance of cotton (*Gossypium barbadense* L.) varieties grown under irrigated and water-limited conditions. Significant differences were observed among the varieties for the traits recorded by the sensors, indicating the reproducibility of the method. Very recently, Phenobot 1.0, a field-based HTPP, equipped with an auto-steered and self-propelled system, has been developed and tested for crops such as sorghum, which have tall and dense canopies (Salas Fernandez et al., 2017). Phenobot 1.0 has RGB cameras that are used to take measurements of plant height and stem diameter very efficiently. The challenge now is to increase the global accessibility of these new technologies.

#### Remote Sensing for High Throughput Phenotyping

Low-cost unmanned aerial vehicles (UAVs) or drones are remote sensing technologies for high throughput phenotyping (HTP) which have already demonstrated significant potential for precision agriculture as they can be used to quantify crop health, and effects of soil moisture and nutrients on crop growth and development (Holman et al., 2016; Khan et al., 2018). These UAVs are capable of providing high resolution images of small experimental plots from distances as high as 30–100 m above ground (Shi et al., 2016; Tattaris et al., 2016). In wheat, aerial measurements of secondary traits such as CT and NDVI, have been successfully used to improve the precision of genomic prediction models for grain yield (Rutkoski et al., 2016). A highly significant association (*r* = 0.76, *p* < 0.05) was observed between vegetative indices extracted from an unmanned aerial system with the ground-truthing data recorded by a spectroradiometer applied to advanced wheat breeding lines (Haghighattalab et al., 2016). Similarly, Tattaris et al. (2016) observed that CT and NDVI measurements in wheat recorded by UAVs were better correlated with yield and biomass compared to the data recorded by proximal measurement under both irrigated and water-limited conditions. Khan et al. (2018) also observed a close association between vegetative indices from aerial images with RGB images in a wheat breeding program. Furthermore, optimized UAVs have been tested in crop growth rate studies. Apart from this, UAVs have been used to assess the growth rate of winter wheat as a response to different rates of fertilizer application (Holman et al., 2016). Very recently, integration of Light Detection And Range (LiDAR), a powerful tool which can acquire precise 3D measurements for crop phenotyping and remote sensing (Madec et al., 2017), has been successfully used in wheat phenotyping studies (Madec et al., 2017; Jimenez-Berni et al., 2018). In China, Crop 3D, an HTP tool developed to integrate LiDAR and UAV, is capable of measuring many plant traits such as plant height, leaf width, leaf length, leaf angle, and leaf area (Guo et al., 2018). Development of improved drought monitoring systems such as the remote sensing drought monitoring system (RSDMS) can have a significant impact on future agricultural research as it was devised based on a model that permits flexibility and increased coverage (Dong et al., 2017). The use of satellite data can also be used to prepare hazard maps that display the distribution of drought risk across a specific region and is being explored for its application to drought tolerance research in crops (Skakun et al., 2016). The pace and progress of new remote sensing technologies potentially offer improvements in selection efficiency, but challenges remain such as poor access to these technologies (e.g., cost) and the need to manage large volumes of data.

## Conclusion and Future Perspectives

In this paper, we have presented evidence-based information related to the negative impacts of drought stress on the productivity of wheat, in the past few decades. Drought affects the physiology of wheat plants resulting in reduced grain yield. The paper highlights the importance of the selection environment for developing high yielding, stable wheat varieties for drought-prone regions. Furthermore, the indirect selection of physiological traits contributing to grain yield shows tremendous potential in terms of improving the efficiency of breeding for drought resilience. Drought may prevail at any growth stage, and the intensity and type of drought can vary for different wheat growing regions. Therefore, here we emphasized the benefits of phenotyping growth stage-based physio- morphological traits. We have also reported the availability of different HTPPs that are capable of measuring these traits.

**FIGURE 5:**
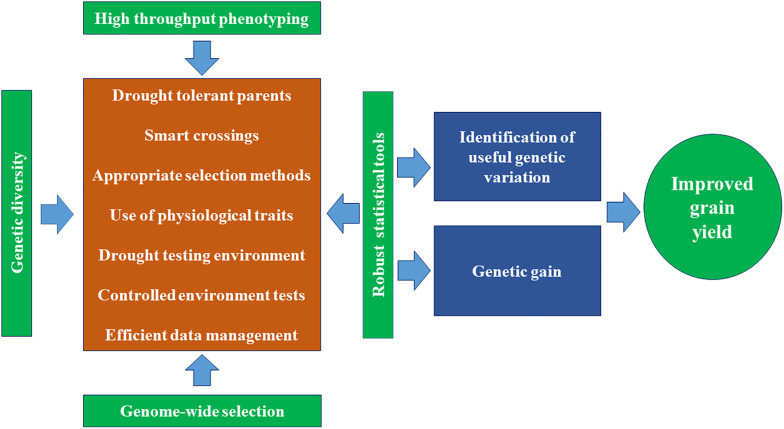
A proposed comprehensive strategy for breeding wheat for drought tolerance.

As we have demonstrated, in many wheat growing environments, the development of drought-tolerant wheat varieties is going to be critical to address the growing food demand. A major challenge associated with the use of physiological traits in plant breeding is that such traits are sensitive to environmental variation (Zarei et al., 2013; Kosová et al., 2014). As this review has highlighted, a multidisciplinary physio-morphological approach is a very promising way forward to enable breeding of wheat varieties for drought-stressed environments (Ortiz et al., 2008). To achieve this goal, a comprehensive effort is needed to establish more efficient platforms for phenotypic selection as described in the sections above, combined with biochemical and marker-assisted and genomic selection. These strategies need to be integrated into a well-organized and comprehensive breeding program (Figure 5) to accelerate the development of new high yielding drought-tolerant wheat varieties. As indicated in Figure 5, utilization of appropriate existing genetic resources, advanced phenotypic and genomic approaches and robust data handling tools potentially improve the efficiency of a breeding program, resulting in a higher genetic gain. To create a breeding program that is capable of generating high yielding and adaptive varieties, smart planning from the beginning is crucial. For example, adopting a “few cross” strategy or smart crossing (Witcombe et al., 2005) could be beneficial; this strategy includes careful selection of parents as per the breeding target and allows opportunities for higher genetic gain. The approach also stresses the benefits of phenotyping physiological traits associated with drought tolerance and testing of breeding materials in diverse environments including under controlled conditions. Dissecting the growth stages further into sub-stages may further improve the genetic gain. As a part of the strategy in Figure 5, data management is also a major part of a breeding program. Newly available open-source software programs have been developed for the benefit of biological research including plant breeding databases such as BrAPI (Selby et al., 2019) and Planteome (Cooper et al., 2018). These programs represent the programming interface for plant breeding and the integrated ontology resource, respectively. The integration of such platforms can improve the adeptness of a breeding program further. The target of the breeding strategy discussed here is to improve the genetic gain while also generating and maintaining new diversity created in the breeding program. In summary, Figure 5 presents an all-inclusive approach for breeding drought tolerant wheat.

Genomic tools such as genome-wide association studies (GWAS), used to identify QTLs associated with physio-morphological traits, benefit substantially from improved phenotypic data. Further adoption of novel molecular technologies such as genome editing (Wang et al., 2018) may open new avenues with respect to breeding for drought tolerance. For example, to enable the development of stress tolerant varieties, Zafar et al. (2020) proposed targeting of regulatory and structural genes responsible for stress tolerance in plants using the CRISPR/Cas9 system. But as has been emphasized, the success of molecular and genomic technologies lies in the quality and quantity of phenotypic data available. In other words, the significance of phenomics is ever increasing. Therefore, more efficient, cost-effective, simple, viable and straightforward platforms for phenotyping morphological, physiological, and phenological traits, and those that reduce the impacts of environmental variation, can have a significant impact on drought tolerance research in wheat.

One challenge that is mounting is the massive volume of data that is generated from high throughput phenotyping (Araus et al., 2018; Yang et al., 2020). This observation highlights the need for platforms to handle the data generated to facilitate the sustained adoption of these techniques. The Minimum Information About a Plant Phenotyping Experiment (MIAPPE) is an example of such platforms that helps in data standardization to promote standard data management practices (Bolger et al., 2019). Similarly, the Consultative Group for International Agricultural Research (CGIAR) centers developed Crop Ontology, a platform to promote proper use of genotypic and phenotypic data through data annotation (Shrestha et al., 2012). However, greater investments are needed to develop tools as well as expertise in bioinformatics for data management and processing along with technologies that offer secure storage of large volumes of data. Training of experts in phenomics, genomics, and data management is as important as technical advancements in these disciplines (Yang et al., 2020). Finally, successful breeding for drought-tolerant wheat varieties will be made possible through collaborations between different institutions or communities across academia, public research institutions, industry, and CGIAR centers. The Heat and Drought Wheat Improvement Consortium (HeDWIC) coordinated by the International Maize and Wheat Improvement Center (CIMMYT), is a great example of a network that includes a wide range of partners and aims to develop climate-resilient wheat varieties. The over 40 year long China-CIMMYT partnership is a notable example of collaboration that has been highly successful in improving wheat (He et al., 2019). However, any such partnerships can only be sustained through long term investments in human resources and visionary project initiatives.

## Author Contributions

KK led the development of this review manuscript. AN, MR, and HE provided the manuscript inputs. MR did the comprehensive editing.

## Conflict of Interest

The authors declare that the research was conducted in the absence of any commercial or financial relationships that could be construed as a potential conflict of interest.
